# Prevalence and Risk Factors of Depression and Anxiety in Essential Tremor Patients: A Cross-Sectional Study in Southwest China

**DOI:** 10.3389/fneur.2019.01194

**Published:** 2019-11-15

**Authors:** Hongyan Huang, Xinglong Yang, Quanzhen Zhao, Yalan Chen, Pingping Ning, Qiuyan Shen, Hui Wang, Ran An, Yanming Xu

**Affiliations:** ^1^Department of Neurology, West China Hospital, Sichuan University, Chengdu, China; ^2^Department of Geriatric Neurology, First Affiliated Hospital of Kunming Medical University, Kunming, China

**Keywords:** essential tremor, depression, anxiety, female sex, cranial tremor, functional disability

## Abstract

**Background:** Essential tremor (ET) is one of the most common movement disorders, and it has been associated with anxiety and depression, but risk factors for these mental symptoms are unclear. This study aimed to evaluate the prevalence and potential risk factors of depression and anxiety in ET patients in China.

**Methods:** This cross-sectional study involved 245 Han Chinese patients with ET. Depression was assessed using the Hamilton Depression Rating Scale-24 Items, and anxiety was assessed using the Hamilton Anxiety Rating Scale. Clinico-demographic characteristics were compared between patients with or without depression or anxiety.

**Results:** We revealed that 63.3% of patients had at least mild anxiety and 54.3% had at least mild depression. Severity of anxiety or depression was correlated with lower Mini-Mental State Exam score (*P* = 0.028 and *P* = 0.002, respectively), higher self-report functional disability score (*P* = 0.027 and *P* = 0.003, respectively), as well as the presence of tremor in the neck (both *P* < 0.001), face (*P* = 0.025 and *P* < 0.001, respectively), or voice (both *P* < 0.001). Binary logistic regression identified female sex, presence with cranial tremor, and greater functional disability score as risk factors of anxiety; the last two factors were also the determinants of depression. Neither anxiety nor depression correlated with physician-assessed severity of arm or leg tremor.

**Conclusion:** Chinese ET patients show high prevalence of depression and anxiety. ET patients should be screened for these mental symptoms, especially women and those with cranial tremor and self-reported functional disability.

## Introduction

Essential tremor (ET) is one of the most common movement disorders, with a prevalence of 4–39 cases per 1,000 worldwide (1). ET is characterized by tremor in upper limbs (including postural, simple kinetic, and intention tremor), and it can involve cranial tremor (including tremor in the neck, face, or voice tremor), as well as non-motor symptoms such as cognitive and mental impairment (2).

ET has been linked to anxiety and depression, with prevalence of these symptoms higher among ET patients than controls (2). In fact, the prevalence of moderate to severe depressive or anxiety symptoms among ET patients can range, respectively, from 5.4 to 48.4% and from 14 to 71.1% (2–6). Depression and anxiety can reduce quality of life (6–8) and self-reported medication compliance of ET patients (8). Depression may even contribute to ET or other important outcomes like embarrassment, rather than simply be a secondary response to tremor symptom (5, 9).

Despite the high prevalence of depression and anxiety in ET and their potential contribution to the disorder, few studies have examined risk factors for these conditions among ET patients. The few available studies have come to conflicting conclusions: some have found an association between tremor severity and depression and/or anxiety in ET, while others have not (2). These discrepancies may reflect the relatively small study populations, as well as differences in how tremor severity and mental symptoms were assessed (3–6, 10–15). ET is not a homogeneous condition: the disease may manifest with different non-motor symptoms and involve different pathological processes depending on the age at onset as well as the type and locations of tremor (16, 17). Studies aiming to clarify potential associations of mental symptoms with ET need to take these issues into account.

As a step in this direction, we undertook the present study with a relatively large sample of Chinese ET patients in order to understand the frequency of depression and anxiety symptoms, their co-occurrence, and the current state of treatment. We also aimed to identify clinico-demographic characteristics that may correlate with depression and anxiety symptoms.

## Method

In this cross-sectional study, a consecutive series of non-demented ET patients was recruited from the Department of Neurology at West China Hospital of Sichuan University (Chengdu, China) between August 2016 and April 2019. None of the patients had ever been diagnosed or treated for ET. The protocol of this study was approved by the Ethics Committee of West China Hospital of Sichuan University. Informed consent was obtained from all participants.

Patients were included if they fulfilled the criteria for probable or definite ET in the consensus statement of the Movement Disorder Society on Tremor (18) based on independent examinations by two neurologists specializing in movement disorders. Patients were excluded if they had the disease <3 years or if they had only isolated neck, face, or voice tremor. Patients were also excluded if they had (a) a medical history of severe head trauma or head surgery; (b) tremor related to other central nervous system disease, such as Parkinson's disease, multiple system atrophy, Wilson disease, or dystonia; (c) tremor related to enhanced physiological tremor, psychogenic tremor, task-specific tremor, orthostatic tremor, drug intake, or alcohol withdrawal; or (d) neuroimaging-based lesions such as infarcts or tumors. These patients were identified based on history and clinical examination.

All patients were interviewed to obtain demographic data and medical history. Demographic details included sex, age at presentation, age at tremor onset, years of education, employment, marital status, current smoking/drinking, disease duration, family history, treatment, medication history, and comorbidities.

### Motor Symptom Evaluation

Severity of rest, postural, and simple kinetic limb tremor was evaluated using the Fahn–Talosa–Marin Tremor Rating Scale (FTM-TRS), a well-validated tremor scoring system (19). The patient-assessed tremor score was obtained using part C of the FTM-TRS, which represents functional disability (19).

#### Action Limb Tremor

Action arm tremor included postural and simple kinetic arm tremor. The score was calculated using items 5–6 in part A of the FTM-TRS (postural and simple kinetic tremor score) as well as part B of the FTM-TRS (19). Action leg tremor including postural and simple kinetic leg tremor was evaluated using items 8–9 on part A of the FTM-TRS (postural and simple kinetic tremor score). If either leg scored at least 1 point, the patient was classified as having action leg tremor.

#### Rest Limb Tremor

Rest limb tremor was assessed using items 5–6 and 8–9 on part A of the FTM-TRS (rest tremor score) (19). Rest tremor was considered presence if the score is at least 1 point in either limb.

#### Intention Tremor

Intention tremor was defined as tremor amplitude that increased during movement close to the target (20). Intention tremor was assessed by the finger–nose–finger test, which involved 10 repetitions per arm (20). Patients were scored as showing no intention tremor (0 point), probable intention tremor (0.5 points), definite intention tremor (1 point), and incapacitating intention tremor (2 points). Patients were classified as having intention tremor if they scored more than 0.5 points in at least one arm or 0.5 points in both arms (20).

#### Cranial Tremor

Cranial tremor included neck, voice, and/or face tremor and the score was evaluated using the FTM-TRS (19). Tremor in each location was present if the individual scored at least 1 point at that location. Patients were classified overall as showing no cranial tremor (0), cranial tremor at one position (1), or cranial tremor at multiple positions (2).

### Cognition and Mental Symptom Evaluation

We screened cognition impairment by using the Chinese version of Folstein Mini-Mental State Exam (MMSE) (21). The presence of depression was assessed using the Chinese version of Hamilton Depression Rating scale-24 Items (HDRS-24) (22). Scores <8 indicated no depression; 8–20, mild depression; and >20, moderate to severe depression (22, 23). Presence of anxiety was assessed using the Chinese version of Hamilton Anxiety Rating Scale (HARS) (24). Scores <7 indicated no anxiety; 7–14, mild anxiety; and >14, moderate to severe anxiety (23, 24).

### Statistical Analysis

Continuous data were presented as mean ± SD and median, while categorical or rank data were reported as numbers and percentages. Inter-group differences in categorical variables were assessed for significance using the chi-squared test. Inter-group differences in continuous variables or rank variables were assessed using the Kruskal–Wallis H test because the data were skewed. *Post hoc* comparisons were Bonferroni-corrected (*P* = 0.017).

Correlations between continuous variables were explored using Spearman's rho correlation coefficient. The correlation coefficient (*r*) value ≤0.19 was defined as a negligible correlation according to the previous report (25). Dependent variables were the absence of depression (HDRS score ≤20) or its presence (HDRS score >20), or the absence of anxiety (HARS score ≤14) or its presence (HARS score >14). We used stepwise binary logistic regression (forward likelihood ratio) to explore risk factors of depression and anxiety in ET. Considering the independent variables had co-linearity and interaction, we included sex, age at presentation, years of education, MMSE score, disease duration, cranial tremor (presence/absence), and self-reported functional disability score (on part C of the FTM-TRS) as independent variables. Correlations were represented as odds ratios (ORs) and 95% confidence intervals (CIs). Statistical analyses were performed in SPSS 23.0 (IBM, Chicago, IL, USA). All tests were two-sided, and significance was accepted at the 5% level (α = 0.05).

## Results

### Demographic and Clinical Characteristics

This study involved 245 patients (125 women, 51.0%) with a mean age at presentation of 53.31 ± 16.44 years and mean disease duration of 9.87 ± 9.74 years. All patients had postural or simple kinetic tremor, while 26 (10.6%) had rest tremor and 78 (31.8%) had intention tremor. All patients had arm tremor; 100 (40.8%), leg tremor; 85 (34.7%), neck tremor; 20 (8.2%), face tremor; and 65 (26.5%), voice tremor. Nearly half the patients (121, 49.4%) had cranial tremor, of whom 79 (32.2%) had cranial tremor at one position, 36 (14.7%) at two positions, and 6 (2.4%) at three positions (Table 1). Treatment of ET is based on the severity of tremor, tremor-related disability, and largely up to the patient's perception of the tremor impact on quality of life. Eventually, 39 (15.4%) patients were treated with β-blockers and 5 (2%) patients were treated with anticonvulsants.

**Table 1:** Demographic and clinical characteristics of 245 patients with essential tremor.

**VARIABLE**	
Age at presentation (years)	53.31 ± 16.44 (55.0)
Female gender	125 (51.0%)
Age of tremor onset (years)	43.40 ± 17.79 (45.0)
Disease duration (years)	9.87 ± 9.74 (5.0)
Education in years	10.64 ± 4.60 (9.0)
MMSE score	27.76 ± 2.23 (28.0)
With family history	103 (42.0%)
With neck tremor	85 (34.7%)
With face tremor	20 (8.2%)
With voice tremor	65 (26.5%)
**Cranial tremor location**	
0	124 (50.6%)
1	79 (32.2%)
2	36 (14.7%)
3	6 (2.4%)
With intention tremor	78 (31.8%)
With rest tremor	26 (10.6%)
With action leg tremor	100 (40.8%)
**With comorbidity**	
Hypertension	26 (10.6%)
Heart disease	5 (2.0%)
Diabetes	8 (3.3%)
Respiratory illness	8 (3.3%)
Osteoarthrosis	17 (6.9%)

*MMSE, Folstein Mini-Mental State Exam*.

*Cranial tremor location defined as 0, without cranial tremor; 1, cranial tremor in one position; 2, cranial tremor in two positions; 3, cranial tremor in three positions*.

### Clinico-Demographic Correlates of Depression and Anxiety in ET

A total of 93 patients (38.0%) had symptoms consistent with mild anxiety, while 62 (25.3%) had symptoms consistent with moderate to severe anxiety (Tables 2, 3). A total of 98 patients (40.0%) had symptoms consistent with mild depression, and 35 (14.3%) reported symptoms consistent with moderate to severe depression.

**Table 2:** Relationship between demographic characteristics and severity of anxiety or depression in 245 patients with essential tremor.

**Variable**	**HARS1 (<7)**	**HARS2 (7–14)**	**HARS3 (>14)**	***P***	***P*1**	***P*2**	***P*3**	**HDRS1 (<8)**	**HDRS2 (8–20)**	**HDRS3 (>20)**	***P***	***P*1**	***P*2**	***P*3**
Number	90 (36.7%)	93 (38.0%)	62 (25.3%)					112 (45.7%)	98 (40.0%)	35 (14.3%)				
Sex (male/female)	56.7%/43.3%	54.8%/45.2%	29.0%/71.0%	0.001^*^	0.803	0.001^*^	0.002^*^	59.8%/40.2%	42.9%/57.1%	31.4%/68.6%	0.004^*^	0.018	0.004^*^	0.315
Age at presentation (years)	53.38 ± 16.54 (56.0)	51.31 ± 18.55 (52.0)	56.21 ± 12.19 (56.0)	0.415				52.52 ± 17.51 (55.0)	52.60 ± 16.59 (54.0)	57.83 ± 11.35 (58.0)	0.372			
Age at tremor onset (years)	44.26 ± 17.50 (46.5)	42.73 ± 19.59 (45.0)	43.16 ± 15.42 (44.5)	0.883				43.61 ± 18.40 (45.0)	42.29 ± 18.00 (44.5)	45.86 ± 15.19 (48.0)	0.617			
Disease duration (years)	9.11 ± 9.00 (4.5)	8.49 ± 7.69 (5.0)	13.02 ± 12.57 (8.0)	0.057				8.86 ± 8.89 (5.0)	10.28 ± 9.42 (6.0)	11.94 ± 12.68 (5.0)	0.167			
Education in years	10.77 ± 4.63 (10.0)	11.23 ± 4.50 (12.0)	9.60 ± 4.62 (9.0)	0.11				10.97 ± 4.56 (12.0)	10.80 ± 4.68 (9.0)	9.17 ± 4.34 (9.0)	0.122			
Employment				0.788							0.301			
Employed	46 (51.1%)	51 (54.8%)	32 (56.5%)					57 (50.9%)	52 (53.1%)	23 (65.7%)				
Unemployed or retirement	44 (48.9%)	42 (45.2%)	27 (43.5%)					55 (49.1%)	46 (46.9%)	12 (34.3%)				
Marriage				0.078							0.318			
Single or divorced	15 (16.7%)	22 (23.7%)	6 (9.7%)					21 (18.8%)	19 (19.4%)	3 (8.6%)				
Married	75 (83.3%)	71 (76.3%)	56 (90.3%)					91 (81.3%)	79 (80.6%)	32 (91.4%)				
With family history	38 (42.2%)	38 (40.9%)	27 (43.5%)	0.945				47 (42.0%)	45 (45.9%)	11 (31.4%)	0.329			
Currently smoking	18 (20.0%)	19 (20.4%)	9 (14.5%)	0.609				25 (22.3%)	16 (16.3%)	5 (14.3%)	0.412			
Currently drinking	14 (15.6%)	11 (11.8%)	9 (14.5%)	0.756				18 (16.1%)	13 (13.3%)	3 (8.6%)	0.570			
With comorbidity														
Hypertension	8 (8.9%)	14 (15.1%)	4 (6.5%)	0.188				13 (11.6%)	9 (9.2%)	4 (11.4%)	0.846			
Heart disease	2 (2.2%)	2 (2.2%)	1 (1.6%)	1.000				2 (1.8%)	3 (3.1%)	0 (0.0%)	0.700			
Diabetes	4 (4.4%)	3 (3.2%)	1 (1.6%)	0.676				5 (4.5%)	2 (2.0%)	1 (2.9%)	0.706			
Respiratory illness	1 (1.1%)	5 (5.4%)	2 (3.2%)	0.285				2 (1.8%)	4 (4.1%)	2 (5.7%)	0.319			
Osteoarthrosis	6 (6.7%)	7 (7.5%)	4 (6.5%)	1.000				8 (7.1%)	6 (6.1%)	3 (8.6%)	0.787			

*Values are mean ± standard deviation (median) or number (percentage)*.

*Abbreviations: HDRS, Hamilton Depression Rating Scale; HARS, Hamilton Anxiety Rating Scale*.

a *Chi-squared or Fisher's exact test*.

b *Kruskal–Wallis H test*. *P1: Pairwise comparisons of variables between (HDRS1 or HARS1) and (HDRS2 or HARS2)*. *P2: Pairwise comparisons of variables between (HDRS1 or HARS1) and (HDRS3 or HARS3)*. *P3: Pairwise comparisons of variables between (HDRS2 or HARS2) and (HDRS3 or HARS3)*.

* *Significant difference*.

**Table 3:** Relationship between clinical characteristics and severity of anxiety or depression in 245 patients with essential tremor.

**Variable**	**HARS1 (<7)**	**HARS2 (7–14)**	**HARS3 (>14)**	***P***	***P*1**	***P*2**	***P*3**	**HDRS1 (<8)**	**HDRS2 (8–20)**	**HDRS3 (>20)**	***P***	***P*1**	***P*2**	***P*3**
Number	90 (36.7%)	93 (38.0%)	62 (25.3%)					112 (45.7%)	98 (40.0%)	35 (14.3%)				
MMSE score^b^	28.11 ± 2.09 (29.0)	27.85 ± 2.11 (29.0)	27.13 ± 2.53 (28.0)	0.028^*^	0.396	0.008^*^	0.059	28.17 ± 2.04 (29.0)	27.71 ± 2.16 (28.0)	26.60 ± 2.67 (27.0)	0.002^*^	0.088	0.001^*^	0.021
With neck tremor^a^	22 (24.4%)	25 (26.9%)	38 (61.3%)	<0.001^*^	0.706	<0.001^*^	<0.001^*^	25 (22.3%)	40 (40.8%)	20 (57.1%)	0.001^*^	0.004^*^	**<**0.001^*^	0.096
Neck tremor score^b^	0.36 ± 0.76 (0.0)	0.47 ± 0.87 (0.0)	1.08 ± 1.03 (1.0)	<0.001^*^	0.474	<0.001^*^	<0.001^*^	0.34 ± 0.75 (0.0)	0.70 ± 0.97 (0.0)	1.06 ±1.06 (1.0)	**<**0.001^*^	0.002^*^	**<**0.001^*^	0.053
With face tremor^a^	6 (6.7%)	4 (4.3%)	10 (16.1%)	0.025^*^	0.705	0.062	0.012^*^	4 (3.6%)	6 (6.1%)	10 (28.6%)	**<**0.001^*^	0.588	**<**0.001^*^	0.001^*^
Face tremor score^b^	0.09 ± 0.42 (0.0)	0.08 ± 0.40 (0.0)	0.23 ± 0.58 (0.0)	0.020^*^	0.736	0.021	0.008^*^	0.05 ± 0.35 (0.0)	0.09 ± 0.41 (0.0)	0.40 ± 0.74 (0.0)	**<**0.001^*^	0.375	<0.001^*^	<0.001^*^
With voice tremor^a^	14 (15.6%)	21 (22.6%)	30 (48.4%)	<0.001^*^	0.227	<0.001^*^	0.001^*^	17 (15.2%)	30 (30.6%)	18 (51.4%)	**<**0.001^*^	0.007^*^	**<**0.001^*^	0.028
Voice tremor score^b^	0.19 ± 0.52 (0.0)	0.26 ± 0.57 (0.0)	0.69 ± 0.97 (0.0)	<0.001^*^	0.349	<0.001^*^	0.001^*^	0.17 ± 0.47 (0.0)	0.38 ± 0.49 (0.0)	0.80 ± 1.08 (0.0)	<0.001^*^	0.018	<0.001^*^	0.013^*^
With cranial tremor^a^	28 (31.1%)	42 (45.2%)	51 (82.3%)	<0.001^*^	0.051	<0.001^*^	<0.001^*^	34 (30.4%)	56 (57.1%)	31 (88.6%)	**<**0.001^*^	**<**0.001^*^	**<**0.001^*^	**<**0.001^*^
Cranial tremor score^b^	0.69 ± 1.40 (0.0)	0.80 ± 1.11 (0.0)	2.03 ± 1.71 (2.0)	<0.001^*^	0.173	<0.001^*^	<0.001^*^	0.60 ± 1.27 (0.0)	1.19 ± 1.35 (1.0)	2.29 ± 1.79 (2.0)	<0.001^*^	<0.001^*^	<0.001^*^	0.001^*^
Cranial tremor grade^b^				<0.001^*^	0.207	<0.001^*^	<0.001^*^				<0.001^*^	<0.001^*^	<0.001^*^	0.002^*^
0	62 (68.9%)	51 (54.8%)	11 (17.7%)					78 (69.6%)	42 (42.9%)	4 (11.4%)				
1	17 (18.9%)	35 (36.6%)	28 (45.2%)					24 (21.4%)	37 (37.8%)	18 (51.4%)				
2	11 (12.2%)	8 (8.6%)	23 (37.1%)					10 (8.9%)	19 (19.4%)	13 (37.1%)				
Action arm tremor score^b^	17.84 ± 8.87 (16.5)	19.30 ± 9.43 (19.0)	19.90 ± 11.54 (16.5)	0.645				17.85 ± 8.51 (17.0)	19.03 ± 10.62 (18.0)	21.26 ± 11.09 (18.0)	0.422			
Action leg tremor score^b^	1.28 ± 1.90 (0.0)	1.41 ± 2.07 (0.0)	1.06 ± 1.72 (0.0)	0.503				1.23 ± 1.89 (0.0)	1.39 ± 2.07 (0.0)	1.11 ± 1.59 (0.0)	0.857			
With action leg tremor^a^	37 (41.1%)	42 (45.2%)	21 (33.9%)	0.374				45 (40.2%)	41 (41.8%)	14 (40.0%)	0.984			
Intention tremor score^b^	0.55 ± 0.61 (0.5)	0.70 ± 0.73 (0.5)	0.76 ± 1.03 (0.5)	0.474				0.55 ± 0.59 (0.5)	0.72 ± 0.89 (0.5)	0.83 ± 0.92 (0.5)	0.528			
With intention tremor^a^	25 (27.8%)	32 (34.4%)	21 (33.9%)	0.581				31 (27.7%)	34 (34.7%)	13 (37.1%)	0.424			
Rest tremor score^b^	0.27 ± 0.94 (0.0)	0.30 ± 1.41 (0.0)	0.29 ± 0.82 (0.0)	0.535				0.31 ± 1.40 (0.0)	0.22 ± 0.73 (0.0)	0.43 ± 1.00 (0.0)	0.150			
With rest tremor^a^	9 (10.0%)	8 (8.6%)	9 (14.5%)	0.490				9 (8.0%)	10 (10.2%)	7 (20.0%)	0.172			
Functional disability score^b^	6.23 ± 4.33 (6.0)	7.12 ± 4.02 (7.0)	8.58 ± 5.64 (7.0)	0.027^*^	0.101	0.008^*^	0.239	6.17 ± 3.90 (6.0)	7.25 ± 4.37 (7.0)	1.09 ± 6.28 (9.0)	0.003^*^	0.084	0.001^*^	0.034

*Values are mean ± standard deviation (median) or number (percentage)*.

*HDRS, Hamilton Depression Rating Scale; HARS, Hamilton Anxiety Rating Scale; MMSE, Folstein Mini-Mental State Exam*.

*Cranial tremor grade defined as 0, without cranial tremor; 1, cranial tremor in one position; 2, cranial tremor in multiple positions*.

a *Chi-squared or Fisher's exact test*.

b *Kruskal–Wallis H test*. *P1: Pairwise comparisons of variables between (HDRS1 or HARS1) and (HDRS2 or HARS2)*. *P2: Pairwise comparisons of variables between (HDRS1 or HARS1) and (HDRS3 or HARS3)*. *P3: Pairwise comparisons of variables between (HDRS2 or HARS2) and (HDRS3 or HARS3)*.

* *Significant difference*.

Univariate analysis showed that severity of anxiety and depression was significantly higher in women as well as in patients with neck, face, voice, or cranial tremor. Higher severity of anxiety and depression was associated with lower MMSE score, higher self-reported functional disability score, and neck, face, voice, or cranial tremor score (Tables 2, 3).

Correlation analysis identified the following scores as associated with HARS score: MMSE (*r* = −0.222, *P* < 0.001), self-reported functional disability (*r* = 0.206, *P* = 0.001), neck tremor (*r* = 0.296, *P* < 0.001), voice tremor (*r* = 0.277, *P* < 0.001), and cranial tremor (*r* = 0.390, *P* < 0.001). The factors were associated with HDRS score: MMSE (*r* = −0.272, *P* < 0.001), self-reported functional disability (*r* = 0.235, *P* < 0.001), neck tremor (*r* = 0.328, *P* < 0.001), face tremor (*r* = 0.204, *P* = 0.001), voice tremor (*r* = 0.269, *P* < 0.001), and cranial tremor (*r* = 0.415, *P* < 0.001). The correlation coefficient between HARS score and HDRS score was 0.855, *P* < 0.001. Scores for neck, face, voice, or cranial tremor did not correlate with arm or leg tremor score.

Binary logistic regression identified female sex, presence of cranial tremor, and higher self-reported functional disability score as risk factors for anxiety in ET patients (Table 4). The latter two factors were also risk factors for depression. Consistent with overlap in risk factors, we identified 30 patients who had concurrent anxiety (HARS score >14) and depression (HDRS score >20), meaning that 48.4% (30 in 62) of the patients with anxiety also had depression and 85.7% (30 in 35) of the patients with depression also had anxiety. Unfortunately, our data did not allow us to determine whether anxiety or depression symptoms occurred first and the sequence in which anxiety and depression occur with tremor.

**Table 4:** Analysis of potential risk factors of anxiety and depression in essential tremor patients.

	**Anxiety**			**Depression**	
**Variable**	**OR (95% CI)**	***P***	**Variable**	**OR (95% CI)**	***P***
Female gender	2.777 (1.391–5.545)	0.004^*^	Cranial tremor	9.507 (3.197–28.275)	<0.001^*^
Cranial tremor	6.601 (3.172–13.735)	<0.001^*^	Functional disability score	1.137 (1.052–1.227)	0.001^*^
Functional disability score	1.098 (1.024–1.178)	0.009^*^			

* *Significant difference*.

Only five patients in our sample had a history of anti-anxiety or anti-depression therapy: one patient was taking benzodiazepines; two patients, a combination of selective serotonin reuptake inhibitors and benzodiazepines; one patient, serotonin and noradrenaline reuptake inhibitor; and one patient, tricyclic antidepressants. For patients with moderate to severe depression and/or anxiety symptoms in our initial evaluation, we recommended them to go to psychiatric outpatient clinics for further assessment, treatment, and to regular follow-up in our department and psychiatric department.

## Discussion

To our knowledge, this is the first large study focused on depression and anxiety in Chinese ET patients. We found that female sex, cranial tremor, and higher self-reported functional disability score may be risk factors of anxiety among these patients, while the latter two parameters may be risk factors of depression. In contrast, physical-evaluated tremor severity in limbs was not a risk factor for either depression or anxiety in these patients.

We assessed anxiety and depression in ET patients by applying the widely used and recognized scales and found that 63.3% of our ET patients had mild to severe anxiety, while 54.3% had mild to severe depression. These prevalences are within the ranges reported in studies from worldwide (2–6). These prevalence ranges are quite broad, which likely reflects differences in the studies, such as whether the sample was patient- or population-based, how large the sample was, and whether mental symptoms were assessed using DSM-IV, or screening scales. These broad ranges may also reflect clinical heterogeneity of ET syndrome and associated comorbidities. In any event, the high frequency of these disorders in ET suggests that they should be considered in ET management strategies. Fortunately, relatively few ET patients seem to suffer moderate or severe depression or anxiety in our sample and in previous studies in China (12).

Despite the high incidence of mental symptoms in ET patients, we found that very few of our patients had been diagnosed or treated for them. Untreated mental disorders correlate with lower quality of life, increased psychiatric disability, and greater use of health care resources (26). Health care workers should consider ET patients to be at elevated risk of depression and anxiety and should screen for these conditions appropriately. In particular, clinicians should be aware of the risk that both depression and anxiety are present, and treatment regimens should be rationally designed.

Cranial tremor and higher self-reported functional disability score emerged as determinants of anxiety and depression among our ET patients. The fact that we assessed all major tremor types and locations as well as the self-reported functional disability score may make our results particularly reliable. This may help clarify controversial results in the literature about associations between tremor severity and depression and/or anxiety in ET. For example, some studies found that depression or anxiety increased with action arm tremor (10, 11), while yet another study did not find that correlation (5). Some studies revealed that depression increased with total FTM-TRS score (including overall tremor and self-reported functional disability score) (12, 13). Another study found that depression, but not anxiety, correlated with FTM-TRS score without the self-reported functional disability component (4, 14).

Our study affirms the value of the self-reported functional disability score for assessing anxiety and depression in ET patients. Consistently, a small retrospective study found that functional disability score on its own was better than physician-reported tremor severity on its own at predicting anxiety and depression in ET patients following deep-brain stimulation (15). The patient-assessed tremor score may capture interaction between functional disability and emotional burden: greater self-perceived functional disability may increase risk of anxiety and depression symptoms, and at the same time, these mental symptoms may amplify subjective feelings of functional disability in daily life. Our results suggest the usefulness of screening ET patients based on functional disability score.

We found that ET patients with cranial tremor were more likely to suffer from depression and anxiety than those without such tremor, regardless of physician-assessed tremor severity at other positions. ET with cranial tremor is known to involve different motor symptom and/or pathology processes than ET without such tremor, such as manifest tandem gait disorder or vermis atrophy (16, 17). In addition to differences in motor symptoms, our study showed that ET patients with cranial tremor also showed differences in non-motor symptoms. At least three considerations support the idea that cranial tremor can increase risk of depression or anxiety in ET. One is that cranial tremor is associated with lesions in the vermis of the cerebellum (17), whereas limb tremor is associated with defects in the cerebellar hemispheres. The lobules VI–VII within the vermis are named the limbic cerebellum and participate in emotion processing, which may partly belong to the salience network and may also be connected with the amygdala and hippocampus; dysregulation in these areas can result in anxiety and depression (27). A second consideration is that balance and gait of ET patients are more severely affected in the presence of cranial tremor, which may exacerbate anxiety and fear (16, 28). A third consideration is that cranial tremor like voice tremor causes communication difficulties that reduce daily living abilities, which may contribute to anxiety and depression. Prospective studies or neuroimaging studies may be needed to further analyze the causes of the co-occurrence of these conditions in the subsequent studies.

Anxiety and depression are risk factors for dementia (29, 30), and our univariate analyses showed that lower MMSE scores were associated with more severe anxiety or depression in our patients. However, no such correlations were detected in multivariate models. These negative results may be considered preliminary because our patients were relatively young and the study did not include patients with dementia.

We found that incidence of anxiety was higher among women than men with ET. This result may reflect gender differences in genetics, psychosocial reactivity, psychosocial gender roles, and emotional expression (31). Clinicians should bear in mind the additional vulnerability of women with ET when performing psychological screening and designing treatment.

While our ET sample was relatively large and we comprehensively assessed potential risk factors of anxiety and depression, our study does have several limitations. The sensitivity of the MMSE scale is relatively lower than that of the MoCA scale, especially in the screening of mild cognitive impairment. Although Hamilton scales have good reliability and validity and are widely used in China, the ability to make a differential diagnosis between anxiety and depression is slightly inadequate. Screening instruments, rather than the DSM-5, were used to assess anxiety and depression, and the cross-sectional design prevents us from concluding any causal relationships. The number of ET cases with moderate to severe depression or anxiety symptom was relatively small and the patients came from one outpatient clinic. Despite this, our sample had adequate power to detect a sizable number of significant associations. Further prospective, longitudinal studies are required to confirm and extend our results.

## Conclusion

Our results illustrate that depression and anxiety symptoms are common in ET. Neurologists should pay more attention to the possibility of these disorders in ET, especially among women and those with cranial tremor and greater self-report functional disability. ET is a heterogeneous syndrome. Our results re-emphasize that patients with cranial tremor may be a subtype of ET.

## Data Availability Statement

The data that support the findings of this study are available from the corresponding author upon reasonable request.

## Ethics Statement

This study was carried out in accordance with the recommendations of the Ethics Committee of West China Hospital of Sichuan University. The protocol was approved by the Ethics Committee of West China Hospital of Sichuan University. All subjects gave written informed consent in accordance with the Declaration of Helsinki.

## Author Contributions

(1) Research project: (A) YX, HH, and XY: conception. (B) HH, XY, QZ, YC, PN, QS, HW, and RA: execution. (2) Statistical analysis: (A) HH, XY, QZ, YC, PN, QS, HW, and RA: design and execution. (B) YX and XY: review and critique. (3) Manuscript: (A) HH: writing of the first draft. (B) YX and XY: review and critique.

### Conflict of Interest

The authors declare that the research was conducted in the absence of any commercial or financial relationships that could be construed as a potential conflict of interest.
